# Corrigendum: Comparison of the frequency and phenotypic profile of *Mycobacterium tuberculosis*-specific CD4 T cells between the site of disease and blood in pericardial tuberculosis

**DOI:** 10.3389/fimmu.2023.1141704

**Published:** 2023-02-06

**Authors:** Elsa Du Bruyn, Sheena Ruzive, Patrick Howlett, Maddalena Cerrone, Ashley J. Jacobs, Cecilia S. Lindestam Arlehamn, Alessandro Sette, Alan Sher, Katrin D. Mayer-Barber, Daniel L. Barber, Bongani Mayosi, Mpiko Ntsekhe, Robert J. Wilkinson, Catherine Riou

**Affiliations:** ^1^ Wellcome Centre for Infectious Disease Research in Africa, Institute of Infectious Disease and Molecular Medicine, University of Cape Town, Cape Town, South Africa; ^2^ Department of Infectious Diseases, Imperial College London, London, United Kingdom; ^3^ Tuberculosis Laboratory, The Francis Crick Institute, London, United Kingdom; ^4^ Center for Infectious Disease and Vaccine Research, La Jolla Institute for Immunology, La Jolla, CA, United States; ^5^ Department of Medicine, University of California San Diego, La Jolla, CA, United States; ^6^ Immunobiology Section, Laboratory of Parasitic Diseases, National Institute of Allergy and Infectious Diseases, National Institutes of Health, Bethesda, MD, United States; ^7^ Inflammation and Innate Immunity Unit, Laboratory of Clinical Immunology and Microbiology, National Institute of Allergy and Infectious Diseases, National Institutes of Health, Bethesda, MD, United States; ^8^ T Lymphocyte Biology Section, Laboratory of Parasitic Diseases, National Institute of Allergy and Infectious Diseases, National Institutes of Health, Bethesda, MD, United States; ^9^ Department of Medicine, University of Cape Town, Cape Town, South Africa; ^10^ Division of Cardiology, Department of Medicine, University of Cape Town, Cape Town, South Africa; ^11^ Division of Medical Virology, Department of Pathology, University of Cape Town, Cape Town, South Africa

**Keywords:** pericardial tuberculosis, site of disease, CD4 response, treatment response, whole blood, pericardial fluid

In the published article, there was an error in the author list, and author Maddalena Cerrone was erroneously excluded. The corrected author list appears above.

The author has also been included in the Author Contributions section shown below.

## Author contributions

CR, EB, DB, KM-B, MN, BM and RW designed the study. PH, MC, AJ and EB recruited the study participants. SR, EB, MC, and CR performed the whole blood and PCF assay. EB and CR performed the flow experiments. CR analysed and interpreted the data. ASe and CLA provided critical reagents. RW, ASh and CR obtained funding to support the project. CR and EB wrote the manuscript with all authors contributing to providing critical feedback.

The authors apologize for this error and state that this does not change the scientific conclusions of the article in any way. The original article has been updated.

