# Supplementary figures and images for: Comparison of the frequency and phenotypic profile of Mycobacterium tuberculosis-specific CD4 T cells between the site of disease and blood in pericardial tuberculosis
Source: Front Immunol. 2022 Nov 11;13:1009016. doi: 10.3389/fimmu.2022.1009016 (PMC9692124; doi:10.3389/fimmu.2022.1009016)

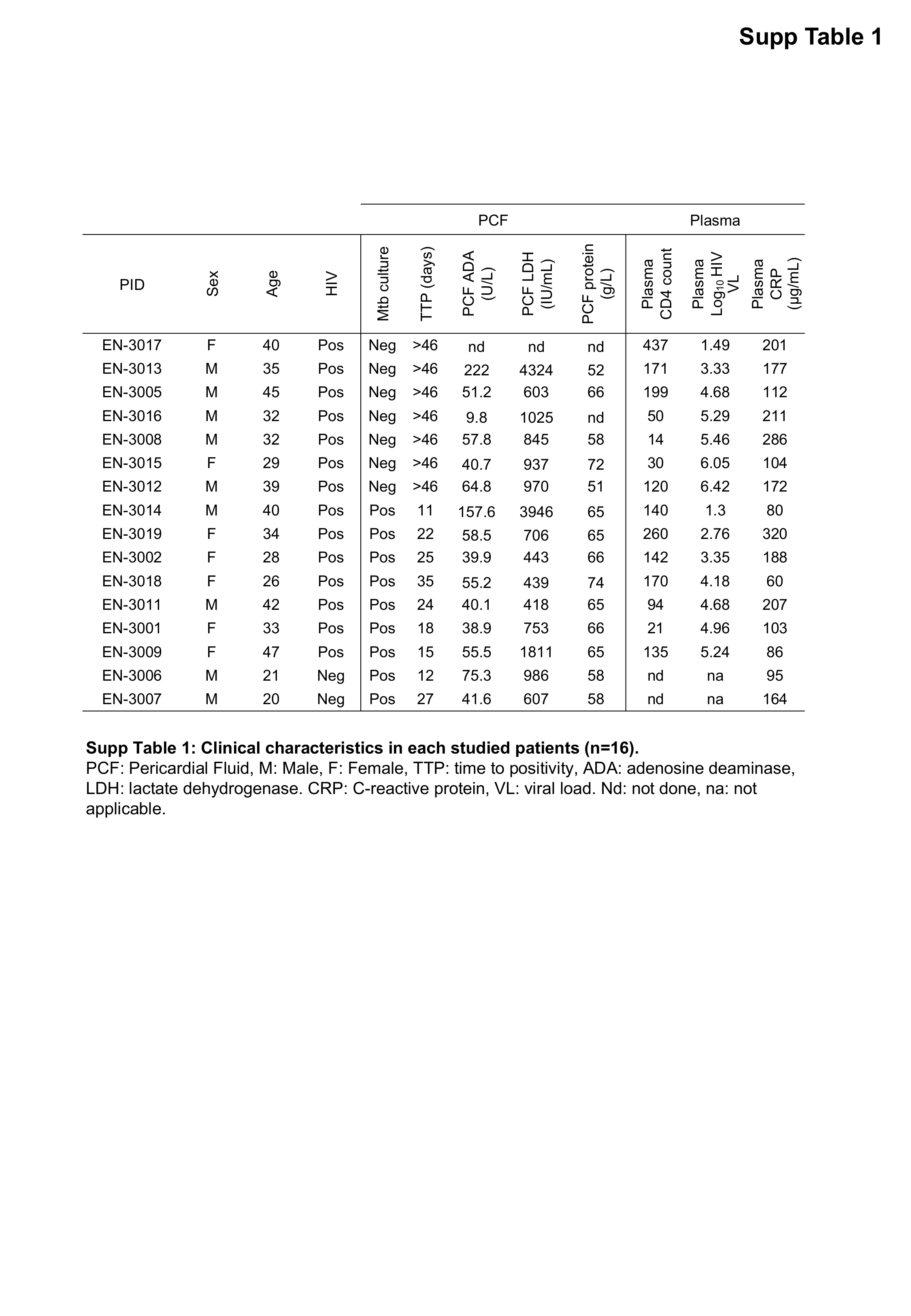

Supplement: Supplementary file 1 [file Table_1.docx]

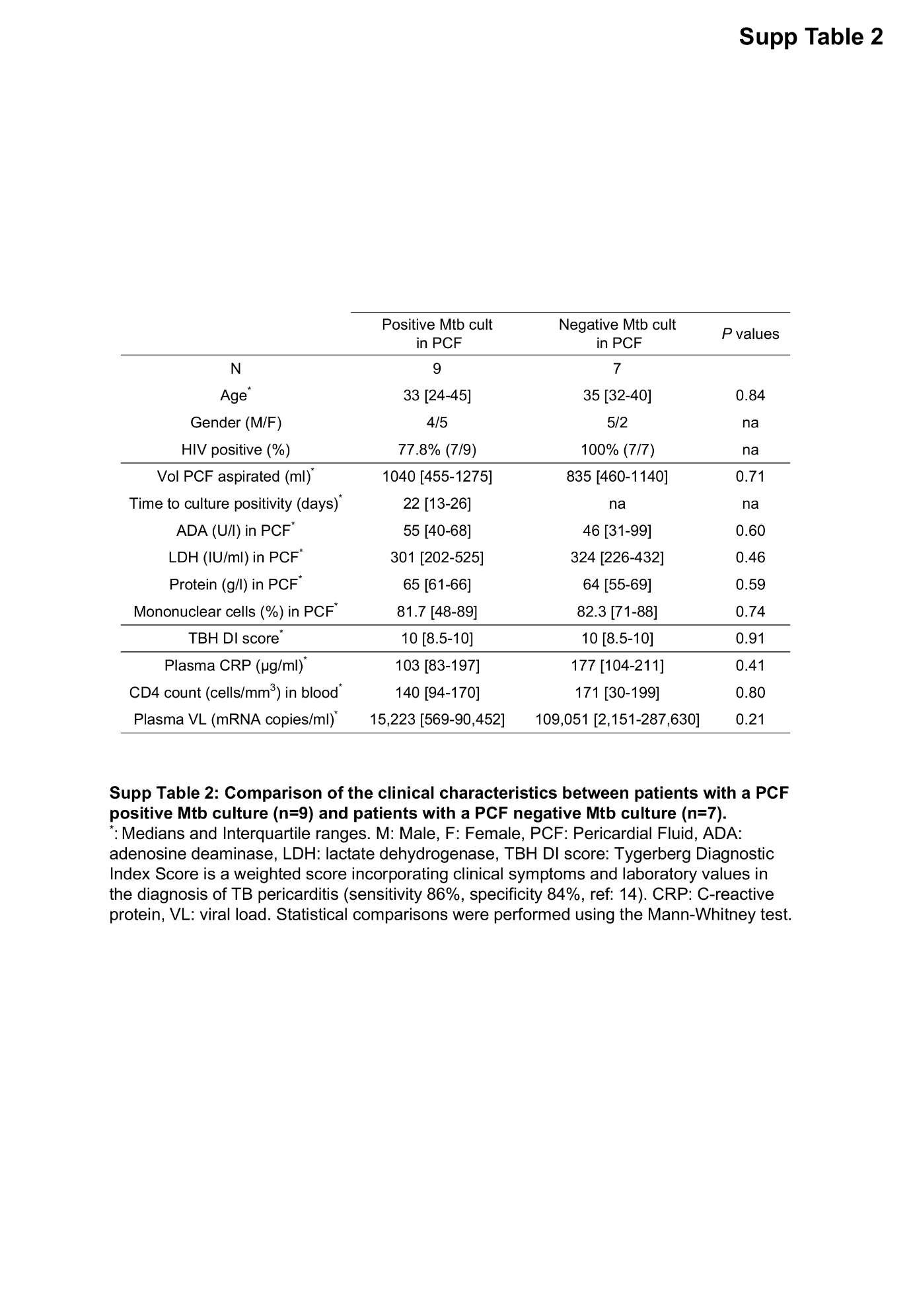

Supplement: Supplementary file 2 [file Table_2.docx]

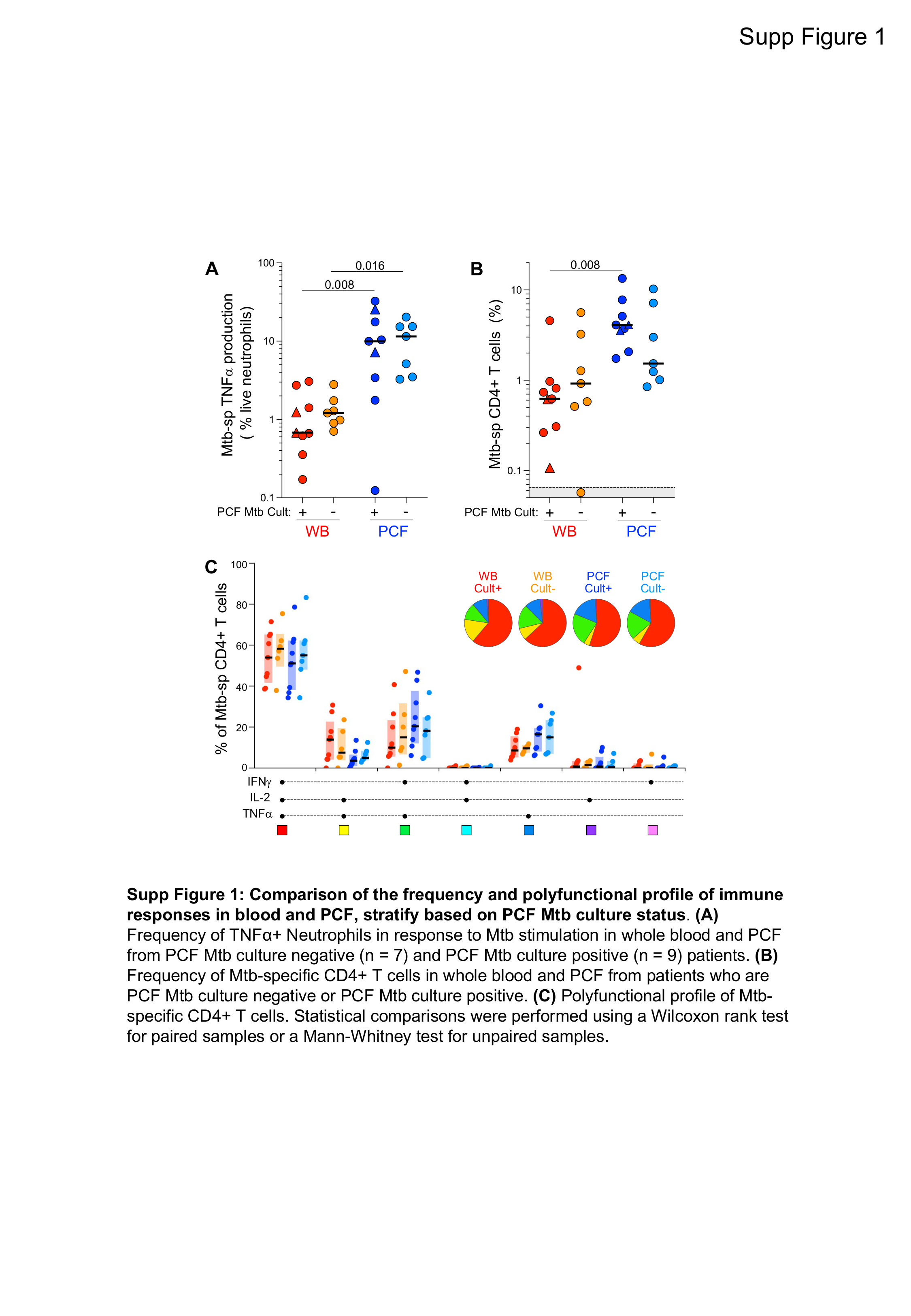

Supplement: Supplementary file 3 [file Image_1.tif]

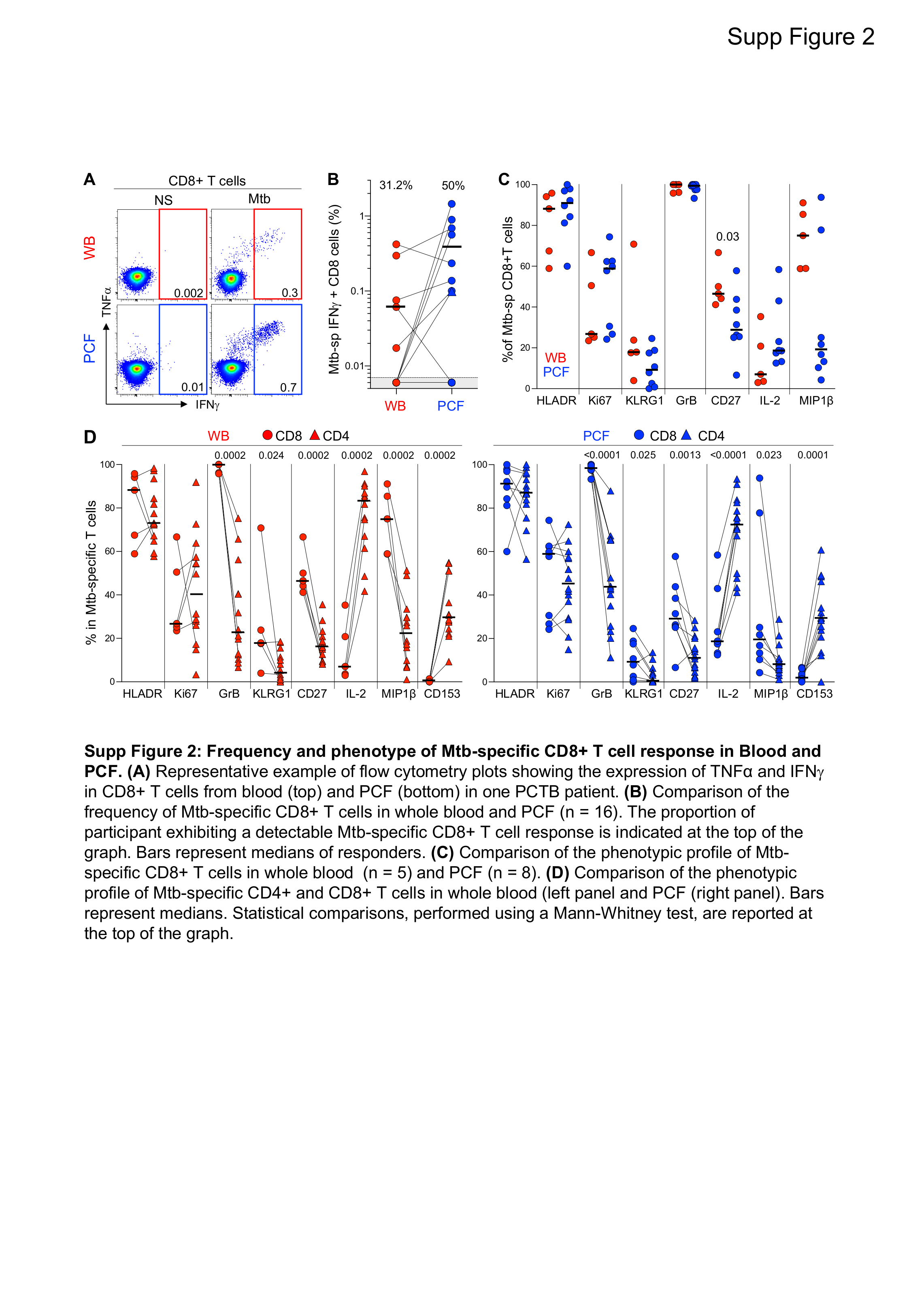

Supplement: Supplementary file 4 [file Image_2.tif]
